# Full-Length TrkB Variant in NSCLC Is Associated with Brain Metastasis

**DOI:** 10.1155/2020/4193541

**Published:** 2020-11-17

**Authors:** Mariangela Lombardi, Michela D'Ascanio, Stefania Scarpino, Davide Scozzi, Marco Giordano, Leopoldo Costarelli, Enrico Rathina Raj, Rita Mancini, Giuseppe Cardillo, Vittorio Cardaci, Marta Innammorato, Andrea Vecchione, Alberto Ricci

**Affiliations:** ^1^Department of Clinical and Molecular Medicine, Sapienza University of Rome, Sant'Andrea Hospital, Rome, Italy; ^2^Division of Cardiothoracic Surgery, Department of Surgery, Washington University, St. Louis, Missouri, USA; ^3^Department of Pathology, San Giovanni Addolorata Hospital, Rome, Italy; ^4^Unit of Thoracic Surgery, Carlo San Camillo Forlanini Hospital, Rome, Italy; ^5^Unit of Pulmonary Rehabilitation, IRCCS San Raffaele Pisana, Rome, Italy

## Abstract

Despite remarkable therapeutic advances have been made in the last few decades, non-small cell lung cancer (NSCLC) is still one of the leading causes of death worldwide. Brain metastases are a common complication of a wide range of human malignancies and in particular NSCLC. Brain-derived neurotrophic factor (BDNF), binding its high-affinity tyrosine kinase B receptor, has been shown to promote cancer progression and metastasis. We hereby investigated the expression of the BDNF and its TrkB receptor in its full-length and truncated isoform T1, in samples from primary adenocarcinomas (ADKs) of the lung and in their metastasis to evaluate if their expression was related to preferential tumor entry into the central nervous system (CNS). By immunohistochemistry, 80% of the ADKs that metastasize to central nervous system expressed TrkB receptor compared to 33% expressing of ADKs without CNS metastasis. Moreover, ADKs with CNS metastasis showed an elevated expression of the full-length TrkB receptor. The TrkB receptor FL/T1 ratio was statistically higher in primary ADKs with brain metastasis compared to ADKs without brain metastasis. Our data indicate that TrkB full-length isoform expression in primary ADK cells may be associated with higher risk to develop brain metastasis. Therefore, TrkB receptor may possess prognostic and therapeutic implications in lung ADK.

## 1. Introduction

NSCLC is an aggressive malignancy with rapid progression and low survival rate [1]. Although the advent of targeted therapies has significantly extended life expectancy, yet only a small percentage of patients are currently eligible. The majority of the advances have been made for the treatment of lung adenocarcinomas (ADKs) which represent the majority of the NSCLC, and that is characterized by considerable molecular heterogeneity [2]. In this regard, different molecular aberrations including EGFR, ALK, and ROS1 mutation have proved to be effective therapeutic targets [3]. Unfortunately, only less than 20% of ADK patients express these sensible mutations, leaving the large majority of NSCLC out of range for a targeted therapy. In addition to that, patients inevitably acquire resistance to this form of treatment over the time [4]. These observations highlight the importance of improving the knowledge of NSCLC molecular biology with the aim of developing novel molecular targets able to extend patient survival.

Neurotrophins (NTs) are a family of growth factors with important pleiotropic function in the central and peripheral nervous system [5]. NT family consists of principally four peptides, the prototype nerve growth factor (NGF), brain-derived neurotrophic factor (BDNF), the NT 3, and the NT 4/5 [6]. All these growth factors bind to the low-affinity pan NT P75 receptor [7]. Moreover, NTs bind specific high-affinity tropomyosin-related kinase (Trk) receptors. BDNF preferentially binds the TrkB, a tyrosine kinase receptor located at the cellular membrane whose intracellular signaling is implicated in neuronal survival and differentiation.

The BDNF/TRKB axis has been shown to be activated in several human malignancies [8, 9] with important effect on cancer cells behavior [10]. In particular, TrkB expression on cancer cells has been associated with aggressiveness and tendency to metastasize [11]. TrkB activate may be expressed in a number of different isoforms [12]. Biological activity required the TrkB full-length isoform (TrkB-FL). The functions of the truncated isoforms (TrkB-T1 and TrkB-T2) are unclear although under some conditions can function as dominant negatives [13]. In addition, the release of BDNF from cancer cells may also be involved in that cross talk between stroma and cancer, facilitating a microenvironment favorable for cancer metastasis. It is well known that microenvironment may be essential for cancer cell survival and development. Based on the well-known neurotrophic function displayed by the BDNF/TRKB axis within the nervous system, we hypothesize that the expression of the TRKB receptors and/or the release of neurotrophic growth factors may allow ADK cells to survive and proliferate in a so specific microenvironment [9]. Furthermore, we have also investigated the possible role of TrkB isoforms to contribute to this phenomenon. Therefore, the aim of the present study has been to demonstrate if TrkB isoforms and BDNF expression in primary ADKs of the lung may be associated with increasing risk to develop metastasis to the brain.

## 2. Materials and Methods

### 2.1. Patients

We collected a total of 55 tissue samples obtained from patients who underwent surgical lung resection or lung biopsies at Sant'Andrea Hospital between 2007 and 2019. All patients had a diagnosis of primary ADK on the histological tissue examination. They were followed clinically and radiologically, for almost two years from the moment of diagnosis. None of them had received treatment or had previous cancer history before lung surgery.

Group 1 was represented by 20 tissue lung samples of patients with brain metastasis. Among them, only 10 patients staged as stages II and IIIa, who underwent lung cancer resection, developed brain solitary metastasis during the follow-up that was treated surgically. Therefore, for these patients, we had the opportunity to process both primitive tumor and the corresponding cerebral metastasis samples. In the same Group 1, we enrolled 10 no resectable ADKs (stages III and IV) with brain metastasis of which we studied the lung cancer specimens.

Group 2 was represented by 15 patients affected with ADK of the lung stages IIIb and IV who did not develop brain metastasis during their follow-up. Samples from these patients were derived from surgical biopsies. These patients were treated with chemotherapy or, in selected cases, by TK inhibitors.

Group 3 was represented by 20 patients affected by ADK of the lung (stages IIIb and IV) with single or polymetastatic disease in site different from the brain. In this group, both lung cancer and metastatic specimens were obtained.

All the samples of each group were processed for TrkB and BDNF immunohistochemical determination.

All patient's characteristics are summarized in Table 1.

The study was approved by the Ethics Committee (protocol number 1032/17).

### 2.2. Cancer Cell Cultures

Established human NSCLC cell line NCI-H460 was used to better clarify the results obtained in human cancer tissues. Cell line was obtained from the American Type Culture Collection (ATCC, Manassas, VA, USA) and was cultured according to the manufacturer's instructions. The cancer cell line H460 was used and grown under aseptic conditions as previously detailed [14]. Briefly, the cells were suspended in RPMI 1640 supplemented with 0.1% bovine serum albumin, 0.5 mg/ml fungizone, 5 mg/ml gentamycin, 5 mM ethanolamine, 10 mM HEPES, 5 mg/ml transferrin, 10 mM T3, 50 mM selenium, 5 mg/ml insulin, and 1 mg/ml hydrocortisone. The cells were cultured at 37°C in culture flasks. All cell culture reagents were obtained from Sigma Chemical Co. (St. Louis, MO).

Cells were grown in adherence (ACC) and in spheroid conditions. To generate spheres, cancer cells were suspended in a Dulbecco's modified Eagle's medium/F12 (Invitrogen Ltd.) supplemented with 50 ng/ml of epidermal growth factor and 25 ng/ml of bovin fetal growth factor. Spheres were observed using a microscope Zeiss Axiovert 25, Jena, Germany. After checking for single cells, the cells were pelleted and suspended in sphere culture medium before replating in nonadherent flasks. In these conditions, cancer cells developed high expression of cancer stem cell-associated markers (CSC) (data not shown).

### 2.3. RNA Extraction, Reverse Transcription, and PCR

The expression of full-length TRKB, TRKB-T1, in lung tissues was studied by evaluating RNA levels. RNA from surgical and biopsy-derived samples was collected from five 5 *μ*m thick sections of tissue mounted on a microscopic slide. The slides were stained in hematoxylin and eosin and evaluated by the pathologist to select areas richer in tumor cells. Total RNA was obtained using the High Pure FFPE RNA Micro KIT (Roche Diagnostics, Germany), an isolation kit designed to extract all nucleic acids (RNA, miRNA, and DNA) from formalin-fixed and paraffin-embedded tissues. TRIzol, phenol, and chloroform were used for RNA extraction from cell lines. RNA integrity was evaluated by agarose gel electrophoresis and spectrophotometry. Four micrograms of total RNA extracted from tissue was reverse transcribed in a final volume of 20 *μ*L using the Fast Gene Scriptase II kit (LS63, NIPPON Genetics Europe, RESNOVA, Italy). The cDNA was at -20°C until further analysis. A positive control also used cDNA obtained from RNA extracted from a sample of diffused cerebral neoplasia. The genes of interest were evaluated by PCR analysis using the following specific primers: TRKB FL forward 5- TAC ATC TGT ACT AAA ATACA-3, reverse 5- GTG TCC CCG ATG TCA TTC GC -3, TRKB T1 forward 5- TAA AAC CGG TCG GGA ACA TC -3 reverse 5- ACC CAT CCA GTG GGA TCT TA -3, and BDNF forward 5- AAC AAT AAG GAC GAC GAC TT -3 reverse 5- TGC AGT CTT TTT GTC TGC CG -3. In order to normalize the amount of total cDNA present in each reaction, the B-actin gene was amplified.

### 2.4. Quantitative Real-Time PCR (qRT PCR)

The expression of full-length TRKB, TRKB T1, in the lung tissues of metastatic and not metastatic patients was studied by evaluating RNA levels using a SYBR Green real-time PCR assay using the following specific primers: TRKB FL forward 5- GGCCCAGATGCTGTCATTAT-3 reverse 5-TCCTGCTCAGGACAGAGGTT-3; TRKB-T1 forward 5-ATCCCTTCCACAGACGTCAC-3 reverse 5- CCATCCAGTGGGATCTTATGAAAC-3; and BDNF forward 5- AACAATAAGGACGCAGACTT-3 reverse 5-TGCAGTCTTTTTGTCTGCCG-3. To normalize the total amount of cDNA present in each reaction, the *β*-actin gene was amplified: actin forward 5- CGGTTCCGCTGCCCTGAG-3 reverse 5- TGGAGTTGAAGGTAGTTTCGTGGAT-3. The data have been expressed as relative units (UR) to control.

Levels of full-length TRKB, TRKB-T1, and BDNF were also measured from 2 stable cell lines derived from lung adenocarcinoma (WT 02/19/18, SPHER-MEP 02/21/18) which differed in the type of growth, adherent or spheroidal.

### 2.5. Immunohistochemistry

Histological sections fixed in formalin and included in paraffin were immunostained with the following antibodies: rabbit anti-human TrkB antibody (sc-12 rabbit polyclonal affinity purified antibody raised against a peptide mapping within the C-terminal cytoplasmic domain of TrkB of mouse origin, Santa Cruz Biotechnology, Dallas, Texas USA) dilution 1 : 500; rabbit anti-human -BDNF polyclonal antibody (H20, sc-548; epitope mapping at the N-terminus of the mature chain of mature NGF of human origin, Santa Cruz Biotechnology, Dallas, Texas USA) dilution 1 : 1000 dilution. After incubation, slides were exposed to anti-rabbit secondary antibody (1 : 100). The product of immune reaction was revealed using the three steps labeled the streptavidin–biotin–immunoperoxidase technique (LSAB2, DAKO, Glostrup, Denmark). The sections were then washed, dehydrated, mounted, and viewed under a light microscope. The analysis of the results was independently performed by two expert pathologists. The staining was evaluated as percentage of positive cells. Cases with more than 5% positive cancer cells were classified as positive. Cases with less than 5% of positive cancer cells were considered negative. The immunohistochemical expression of the TRKB and BDNF protein was also evaluated as color intensity (1, low intensity; 2, medium intensity; and 3, high intensity). No specific binding was obtained by antibodies preabsorbed with the corresponding blocking peptides (10 *μ*g/ml).

### 2.6. Statistics

Data were expressed as mean ± standarddeviation (SD). The chi-square test or Fisher's exact test was used for categorical data, and Student's *t*-test was used for continuous data. Statistical analysis was performed by using GraphPad, and a *p* value < 0.05 was considered significant. All in vitro experiments were performed in triplicate.

## 3. Results

### 3.1. Immunohistochemical Expression of TrkB and BDNF

**Figure 1:**
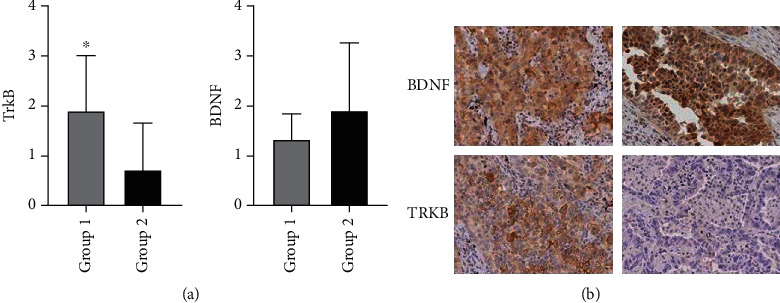
Immunohistochemical detection of TrkB and BDNF protein expression in sections of primitive ADKs of the lung with (Group 1) or without (Group 2) brain metastasis. The histograms summarize the results obtained. A statistically significant increase of TrkB protein expression was observed in samples from primitive ADK in Group 1 compared with Group 2. The BDNF protein expression was higher in Group 2 in comparison with Group 1. Notice the anatomical localization of BDNF and TrkB protein expression in ADK cell. BDNF antibodies generated a prevalent nuclear dark brown immunoreaction. The TrkB antibody generated a cytoplasmic and cell membrane immunoreaction. A clear and specific TrkB and BDNF immunostaining was documented in ADKs cells in both primitive and brain metastasis in Group 1. Contrarily, in Group 2, a strong and specific BDNF immunostaining was documented in brain metastasis, but not for TrkB. The data are the mean + SD of different experiments performed in triplicate. ×20 ∗*p* < 0.0177.

**Figure 2:**
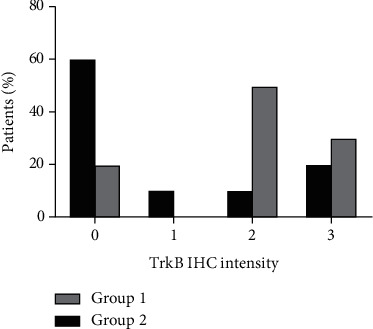
Distribution among the groups of TrkB intensity immunostaining (0: expression < 5%; 1: low intensity; 2: moderate intensity; and 3: high intensity) detected in sections of primitive ADKs of the lung with (Group 1) and without (Group 2) brain metastasis. The results are the mean of three independent expert pathologist evaluation. ×20.

The expression of TrkB was demonstrated by immunohistochemistry (IHC). The expression of TrkB was detected in paraffin-embedded section obtained from samples of lung cancer and brain, skin, bone, and liver metastases. The reaction product was visualized in brown in tumor immunopositive cells, within the cytoplasm. The results showed a statistically significant difference, in the TrkB receptor immunohistochemical expression, in tissue samples obtained from ADKs of the lung obtained from patients belonging to Group 1 (lung cancer with brain metastasis) and Group 2 (lung cancer without brain metastasis) (1.9 ± 1.1 and 0.7 ± 0.9, respectively) expressed in arbitrary unit (*p* = 0.017) (Figure 1). On the contrary, the expression of BDNF is higher in the Group 2 (1.33 ± 0.5 in Group 1; 1.88 ± 1.3 in Group 2) (*p* = 0.26) (Figure 1). The number of patients that expressed TrkB in medium-high intensity in Group 1 versus Group 2 was, respectively, 16/20 (80%) and 5/15 (33%) (Figure 2).

**Figure 3:**
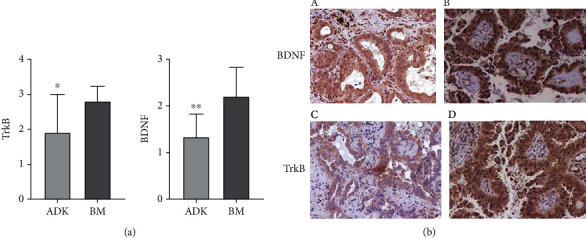
Immunohistochemical detection of TrkB and BDNF protein expression in section samples obtained by Group 1 (ADK with brain metastasis). (a) The histograms summarize the results obtained. A statistical significant increase of TrkB and BDNF protein expression was observed in samples from metastasis compared with relative primitive ADKs. (b) Notice the anatomical localization of BDNF and TrkB protein expression in ADK cells. A strong specific immunostaining was clearly documented in both primitive ADKs and brain metastasis (a–d). The data are the mean ± SD of different experiments performed in triplicate. ADK, primitive ADKs; BM, corresponding brain metastasis. ∗*p* < 0.02; ∗∗*p* < 0.004.

**Figure 4:**
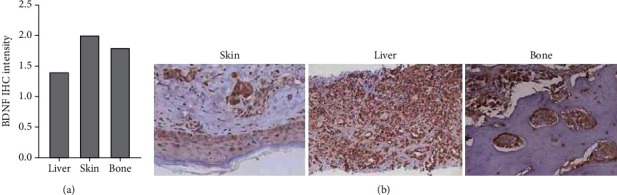
Immunohistochemical detection of BDNF protein expression in section of metastasis from the skin, liver, and bone. The BDNF immunostaining was strongly expressed in the different metastatic sites studied (a, b). Note the specific clear immunostaining within cancer cells in metastatic cancer cells. ×20.

TrkB and BDNF were also demonstrated by IHC in patients belonging to Group 1 in both lung cancer and the corresponding brain metastasis. The expression of TrkB was higher in brain metastasis than corresponding primitive lung cancer (2.8 ± 0.4 vs. 1.9 ± 1.1) (*p* = 0.02) (Figure 3). Similarly, BDNF was more expressed in metastasis than in the corresponding primitive lung cancer from which they originated (2.2 ± 0.6 vs. 1.3 ± 0.5) (*p* = 0.004) (Figure 3). Finally, the expression of TrkB and BDNF was estimated on sample tissue obtained from metastasis of lung cancer different from brain, in particular, liver (*n* = 13), bone (*n* = 2), and skin (*n* = 5) (Figure 4). In these samples, BDNF antibody generated a specific immunostaining in cancer cells in the 75% of the samples. On the contrary, we detected a specific TrkB immunostaining in only one sample of liver metastasis.

### 3.2. Real-Time Quantitative Reverse Transcription PCR

**Figure 5:**
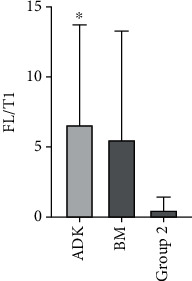
qRT-PCR. The histogram emphasizes the elevated TrkB FL/T1 ratio in primary ADKs of the lung with brain metastasis. The data are the mean ± SD of different experiments performed in triplicate. ADK, primitive ADKs; BM, corresponding brain metastasis. ∗*p* < 0.01.

Since our immunohistochemical analysis was unable to discriminate which receptor isoform (full-length TRKB or TRKB-T1) prompted us to identify the relative expression of the different isoforms by further and more sensitive investigation techniques. qRT-PCR allows us to enable reliable detection and measurement of products generated during each cycle of PCR process. Thus, we demonstrated the expression of the full-length (FL) TrkB receptor and truncated (T1) isoforms. The expression of the FL is higher in Group 1 compared to Group 2 (1.025 ± 0.68 and 0.5 ± 0.96, respectively). Contrarily, the T1 isoform was more expressed in Group 2 (Figure 5). Interestingly, the FL/T1 ratio confirmed this behavior, showing that patients belonging to the Group 1 had a statistically higher ratio in comparison with Group 2 (6.59 ± 7.10 vs. 0.48 ± 0.94) (*p* = 0.014) (Figure 5).

### 3.3. TrkB Receptor and BDNF in Primary Adherent and Spheroid Cell Cultures

Spheroid cancer cell mimics the characteristics of cancer stem cells with migratory capacity and resistance to chemotherapy resembling the “in vitro” counterpart of metastatic cells [15].

**Figure 6:**
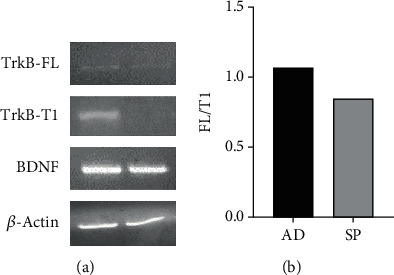
RT-PCR (a) and qRT-PCR (a) to clarify the FL TrkB and TrkB-T1 isoform expression in lung adenocarcinoma cell lines (NCI-H460) grown in adherence (AD) or in spheroid conditions (SP). The total amount of cDNA present in each reaction was normalized by *β*-actin gene amplification. (a) The RT-PCR display and increased expression of the FL/T1 ratio in spheroid cancer cell. This data was confirmed by qRT-PCR (b). AD, adherent cell lines; SP, spheroid cell lines.

For this reason, we compared the TrkB and BDNF levels in spheroid versus adherent cancer cells. Data obtained by RT-PCR and qRT-PCR showed that the expression of both isoforms was higher in cell grown in adherence. Moreover, the FL/T1 ratio was higher in cell grown under spheroid conditions (1.07 ± 0.05 and 1.14 ± 0.02 in three independent experiments) (*p* < 0.05) (Figure 6).

## 4. Discussion

Lung cancer is the leading cause of cancer-related death worldwide. Brain metastasis occurs in almost 40% of NSCLC patients [16] and is associated with a poor prognosis [17]. However, the factors that can favor brain metastasis in NSCLC are still poorly understood. Here, we show that patients affected with ADK express higher levels of TrkB in the presence of brain metastatic disease.

In the last few decades, it has become increasingly clear that NTs and their TrkB receptors have a wide range of biological effects in different human malignancies [9, 18]. In the lung, NT and their high-affinity receptors have been shown to be present in tumor cells [18]. Moreover, the TrkB receptor expression has been often associated with unfavorable prognosis [19]. Furthermore, we previously demonstrated that TrkB expression in cancer cells promotes epithelial to mesenchymal transition and that its inhibition results in a less-aggressive phenotype [20]. Taken together, these observations point to TrkB/NT axis as a promising target for the treatment of lung cancer.

TrkB possesses at least 3 splice variants: the catalytic tyrosine kinase receptor and two different isoforms that lack the intracellular kinase domain [21] named T1 and T2. Usually, T1 and T2 receptors are able to attenuate or inhibit full-length activation when coexpressed with the full length [22].

To this end, coexpression of BDNF and TrkB is often responsive of poor prognosis and cancer progression and migration in several tumor types [23]. Moreover, T1 expression in squamous cell lung carcinomas displayed better outcomes. Our data firstly show that ADKs with brain metastasis are more likely to express FL TrkB then the truncated isoform. This data would confirm the inhibitor role for TrkB-T1 receptor, while the metastatic involvement of the CNS appears to be a more frequent complication in patients with the exclusive or prevalent full-length TrkB expression. The TrkB FL/T1 ratio may be a potential feature to hypothesize the possible risk to develop CNS metastasis.

The BBB is a complex anatomical barrier, and the mechanism by which cancer cells pass through is still debated [22]. It is in fact known that the blood brain barrier (BBB) remains partially intact in experimental brain metastases [24]. Adhesion interaction between metastatic cancer cell and activated endothelial cells mediated by integrins and vascular cell adhesion molecules (in particular VCAM-1) upon activation by inflammatory stimuli may represent a key mechanism [22].

Our data show that BDNF levels are increased in ADK with brain metastasis. Interestingly enough, BDNF has been previously shown to be the ligand for the alpha9beta1 integrin whose activity could be related to its high cross-reactivity with a variety of endogenous ligands such as VCAM-1, tenascin C, and osteopontin [25–29]. The binding of BDNF with this receptor displays complex proliferative and migratory activity and may potentially promote migration through the BBB [30]. Moreover, among factors that promote vascular proliferation and neoangiogenesis, BDNF has been shown to increase VEGF release enhancing angiogenesis through a signal transduction pathway that involves the TrkB receptor and the hypoxia-inducing factor- (HIF-) 1*α* expression [31].

Contrarily to primary cancer cells, in brain metastasis, we have documented an increasing expression of BDNF, which may be responsible for favoring cancer engraftment and metastatic progression. It is known that disseminated cancer cells derived from primary tumor, possessing a mesenchymal phenotype, once engrafted into the brain, may undergo a reverse process known as mesenchymal to epithelial transition; the transition actions for EMT and MET should be critical to metastasis or demetastasis process [32]. This phenomenon may explain the different TrkB receptor and BDNF expression observed between primary and metastatic cancer cells derived from the same patients. BDNF may be extremely important for the survival of metastatic cancer cells within the central nervous system. It is important to underline that metastases into CNS are preferentially located in the brain areas in which higher levels of BDNF are demonstrated. Interestingly, in our cases, brain metastases are mainly located in the brain areas request in BDNF like frontal and parietal cortexes [17].

Although our data are based on a small-case series, it may stimulate further analysis for possible therapeutic application.

## 5. Conclusion

In ADKs of the lung, TrkB receptor expression in its FL isoform is often associated with metastatic brain disease. On the contrary, the T1 truncated isoform share a protective role. The TrkB FL/T1 ratio determinate in primary ADKs of the lung may be considered a potential prognostic factor able to predict the risk to develop CNS metastasis.

## Figures and Tables

**Table 1:** Demographic and clinical characteristics of patients studied.

Patients	Group 1 (*n* = 20)	Group 2 (*n* = 15)	Group 3 (*n* = 20)
Age	62.90 + 7.6	73 + 4.2	61 + 15.75
Gender (F/M)	6/14	11/9	12/8
Stage at diagnosis	II-III: 10 IIIb, IV: 10	III: 8 IV: 7	III: 12 IV: 8
Lung cancer (ADKs)	20	15	20
Brain metastasis	10	nd	nd
Bone (2), skin (5), and liver (13) metastases	nd	nd	20

nd: not detected.

## Data Availability

The data used to support the findings of this study are available from Lombardi Mariangela upon request.
